# Treatment of obesity in spinal cord injury with tirzepatide: a case report

**DOI:** 10.1038/s41394-025-00699-w

**Published:** 2025-03-01

**Authors:** Michael Juszczak, Kazuko Shem

**Affiliations:** 1 Reading Hospital Rehabilitation at Wyomissing, Tower Health Reading, Wyomissing, PA USA; 2 Santa Clara Valley Medical Center, San Jose, CA USA

**Keywords:** Obesity, Translational research, Weight management

## Abstract

**Introduction:**

Individuals with spinal cord injury (SCI) experience alterations in metabolism that result in increased central obesity, insulin resistance, and dyslipidemia placing them at elevated risk for developing cardiometabolic disease (CMD). Increased exercise and dietary modifications are the primary interventions for preventing CMD. However, people with SCI face unique challenges that prevent them from increasing their physical activity and easily modifying their nutritional intake. Tirzepatide is a medication that has been approved by the Food and Drug Administration to be used in conjunction with lifestyle changes to treat obesity in adults with type 2 diabetes mellitus.

**Case presentation:**

A male in his 40’s with C6 American Spinal Injury Association Impairment Scale B SCI 15 years prior with a body mass index of 32 presented to his primary care provider for treatment of obesity. He previously worked with multiple dietitians and increased his physical activity to lose weight. Despite these interventions, he was unable to reduce his weight. He was started on tirzepatide. After 3 months of treatment, he lost 31 pounds and saw improvements in his lipid profile. The only adverse effect reported was heartburn.

**Discussion:**

The metabolic dysfunction associated with SCI and barriers to adequate exercise for weight loss place individuals with SCI at increased risk for obesity and developing CMD. Tirzepatide may be an effective adjunct therapy to lifestyle interventions to help prevent CMD in those with SCI. Further research is indicated to examine the long-term efficacy, benefits, and adverse effects that may be associated with tirzepatide.

## Introduction

In the United States, it is estimated that 300,000 persons are living with spinal cord injury (SCI). While mortality from respiratory illness, urological diseases, and infections have declined, cardiovascular disease (CVD) has become a leading cause of death among individuals with chronic SCI [1–4]. Persons with SCI have a higher risk of mortality compared to individuals of the general population, with a two to five times higher likelihood of premature death, with CVD causing approximately 35% of deaths among people with SCI who are over the age of 60 and 46% of all deaths among individuals who are more than 30 years old post-injury [3, 5]. The premature development of CVD in those with SCI is thought to be due to multiple risk factors that have been linked to the early development of cardiometabolic disease (CMD) [6–8]. These CMD risk factors include central obesity, insulin resistance, hypertension, atherogenic dyslipidemia, and elevated levels of inflammatory markers, such as high-sensitivity CRP (hs-CRP) [6, 9].

Clinical practice guidelines published by the Consortium for Spinal Cord Medicine (CSCM) have found obesity to be the most prevalent risk factor associated with the development of CMD [10]. Central obesity is the accumulation of adipose tissue around the waist and is defined as a waist circumference greater than 35 inches for women and 40 inches for men in the general population [9]. Compared to those without SCI of the same weight, persons with SCI have a greater incidence of central obesity [11, 12]. A higher prevalence of dyslipidemia, characterized by low levels of high-density lipoprotein cholesterol (HDL-C) and an elevated ratio of total cholesterol (TC) to HDL-C is thought to act synergistically with increased central obesity to accelerate the development of CMD among individuals with SCI [13, 14]. Higher levels of visceral adipose tissue (VAT) promote chronic systemic inflammation by increasing the release of proinflammatory adipokines and thus contributing to the development of CMD in those with SCI [15]. Those living with SCI are also at increased risk for developing insulin resistance and glucose intolerance, both of which can lead to increased VAT and the development of CMD [16].

Many studies have associated the prevention and amelioration of CMD with improved quality of life [17–19]. Despite the emphasis placed on mitigating CMD risk factors among those living with SCI, the literature examining dietary intervention in this population is scarce. A recently published systematic review found only eight studies examining the impact of dietary interventions on mitigating CMD risk factors in those with SCI [20]. A different systematic review found that those living with SCI consume more food than is needed to sustain their basal metabolic rate (BMR), which results in an energy imbalance and increased adiposity [21].

Exercise has been shown to reduce the risk of developing CMD; however, individuals with SCI encounter unique barriers to exercise that those of the general population do not experience such as lack of access to appropriate exercise equipment and specialized facilities to accommodate people living with SCI [22, 23]. When those living with SCI do participate in exercise programs, they often engage in suboptimal levels of physical activity that is not strenuous enough to compensate for their excessive caloric intake [24]. Consequently, the CSCM has suggested that nutritional intervention may be a more practical means for mitigating the development of CMD in those with SCI [10]. Despite making adequate dietary changes and adhering to an exercise program following SCI, mitigating the development of CMD may still be limited by an overall reduction in body weight and VAT [25].

Medications that have been approved by the Food and Drug Administration (FDA) for the treatment of diabetes have also been shown to result in weight loss [26]. Naltrexone-buproprion, phentermine, liraglutide, and lorascerin have all been associated with a significant body weight reduction when continued for 52 weeks total [26]. Each of these medications has different mechanisms of action (MOA) that can lead to unique adverse events, which ultimately do not warrant them to be used for generalized weight loss purposes. For those living without SCI, clinical trials evaluating the efficacy of anti-obesity medications have shown a 5–22.5% body weight reduction when combining pharmacological intervention with lifestyle modifications [26].

In May of 2022, the FDA approved the use of tirzepatide to use in conjunction with lifestyle changes to facilitate weight loss and improve glycemic control in adults living with type 2 diabetes mellitus (Type 2 DM). Tirzepatide is a glucagon-like GLP-1 and glucose-dependent insulinotropic polypeptide (GIP) agonist that has been shown to have a dose-dependent effect on weight loss and lowering HbA1c [26]. A study of 2519 participants who did not have DM found that those administered tirzepatide 15 mg weekly over 72 weeks on average lost 18% of their baseline body weight compared to those who received placebo [14]. The long-term efficacy of tirzepatide is also well studied, with one trial showing that those who continued to take the drug one year after completing treatment reported significant weight loss compared to those who stopped taking the drug [27]. A higher dose of tirzepatide over 42 weeks of treatment has also been shown to lead to greater reductions in body weight, reductions in CMD risk factors, and improved glycemic control and lipid panels [28].

This case report examines the use of tirzepatide to treat neurogenic obesity refractory to dietary and lifestyle intervention in a man living with chronic SCI.

## Case description

A Caucasian man in his late 40’s who sustained a C6 American Spinal Injury Association Impairment Scale (AIS) B SCI 15 years ago due to a mountain bike accident presented to his primary care provider (PCP) for management of obesity diagnosed two years prior. With the patient’s permission, information on this case report was extracted from his electronic medical records, and additional information was obtained from the patient (i.e. self-report). His past medical history (PMH) included neurogenic bladder, autonomic dysreflexia, spasticity and a colostomy (done two years prior). Notably, the patient did not have a PMH significant for Type 2 DM. Medications he has routinely taken over the years only included oxybutynin for bladder relaxation.

Over the previous 5 years he had worked with multiple registered dietitians (RD) and was previously prescribed phentermine to assist with weight loss, though this was ineffective. The patient had completed metabolic testing using VO2 max test, which estimated he must consume 2400 kcal per day to meet his BMR. His RD placed him on a daily 1600 kcal diet consisting of 40% carbohydrates, 30% fat, and 30% protein for three months. He had kept a diet log, and despite consuming 800 kcal below his BMR, he was unable to lose weight. His caloric intake was decreased to 1350 kcal per day, but he was still unable to lose weight. His exercise program for the last five years consisted of upper extremity weightlifting five times per week for 1 h and 15 min per session at a self-perceived exertion level of 7–8 out of 10. He also completed 1 h of modified mountain bike hand cycling at least twice per week.

An adult weight management specialist initiated treatment with tirzepatide as an adjunct therapy to dietary intervention and his exercise program. Before treatment, the patient weighed 115.7 kg (255 lbs) and was 190.5 cm (6′3″) tall with a body mass index (BMI) of 31.87. Relevant laboratory studies obtained before initiating treatment included a glucose of 82 mg/dL and a hemoglobin A1C of 5.3%. The most recent lipid panel was obtained two years prior and is shown in Table 1.

**Table 1:** Changes in lipid profile.

Component of Lipid Profile	2 Years Prior to Treatment	2 Months after Initiating Treatment
Total Cholesterol (mg/dL)	228	203
Triglycerides (mg/dL)	143	130
HDL Cholesterol (mg/dL)	41	38
LDL Cholesterol (mg/dL)	158	139
Cholesterol to HDL Ratio	5.6	5.3

The patient was initially started on tirzepatide 2.5 mg injections weekly by his adult weight management specialist who placed him on an automatic 20-week dose escalation program with tirzepatide. After 1 month of treatment, his weight decreased to 112.5 kg (248 lbs) (see Table 2). During his second month of treatment his dose was increased to 7.5 mg weekly, which resulted in his weight further decreasing to 111 kg (244 lbs). His dose was again increased to 15 mg weekly during his third month of treatment. During his third month of treatment the patient sustained a thermal injury to his right buttocks while wearing heated pants for pain which resulted in hospitalization. While hospitalized, he underwent a skin flap procedure to facilitate proper wound healing. At his request, he elected to temporarily stop treatment with tirzepatide while hospitalized. While hospitalized he was evaluated by an RD who placed him on a standardized diet of 2100–2300 kcals per day, consisting of 130–150 grams protein/day, and 2000–2100 mL fluid/day. In total, he was hospitalized for 1 month. Despite not receiving tirzepatide injections while hospitalized, his weight continued to decrease. On the day of discharge his weight was recorded as 101.6 kg (224 lbs). He resumed treatment with tirzepatide after being discharged home and continues to receive 15 mg injections weekly. After one year of treatment with tirzepatide his weight was recorded as 100.7 kg (222 lbs) at a follow-up appointment with his PCP. In total, he lost 14.9 kg (33 lbs) after one year of treatment. The only adverse effect he reported throughout his treatment was mild heartburn. His overall change in body weight throughout his treatment course can be seen in Table 2.

**Table 2:** Change in bodyweight throughout course of treatment with tirzepatide.

Time Point	Body Weight (kg/lbs)
Day prior to starting tirzepatide	115.7/255
1 month after starting tirzepatide	112.5/248
2 months after starting tirzepatide	111/244
3 months after starting tirzepatide	104/229
Admission to hospital for thermal injury	104/229
At discharge from hospital for thermal injury	101.6/224

## Discussion

Prior literature suggests increased prevalence rates of developing CMD following SCI (25 to over 50%) when compared to those among similarly aged individuals of the general population [29–31]. A recently published study found that one third of patients discharged from acute rehabilitation after SCI had a poor cardiovascular risk profile suggesting that changes in body composition as a result of metabolic dysfunction after SCI begin to manifest as early as discharge from acute rehabilitation, which emphasizes the importance of early intervention to mitigate the development of CMD [32].

Current literature surrounding dietary intervention to prevent neurogenic obesity in SCI is sparse and often indicates that dietary intervention alone is not sufficient to reduce weight in those with SCI. There are even fewer studies examining the impact of dietary intervention on reducing risk factors that lead to the development of obesity related CMD [33]. Therefore, exploring the use of pharmacological interventions as an adjunct therapy to dietary and exercise interventions may be reasonable for those who are unable to achieve weight loss. As supported by our case study, utilizing tirzepatide in conjunction with dietary modification and increased physical activity may be an effective intervention to assist those unable to lose weight.

Previous studies in the general population have noted improvements in waist circumference, blood pressure, fasting insulin levels, lipid levels, and the ratio of total fat mass to lean fat mass in those receiving tirzepatide to assist with weight loss [34]. All of these variables have been identified as associated factors leading to the development of CMD. In addition to losing a significant amount of body weight and lowering his BMI, our patient also saw improvements in his lipid profile just two months after initiating treatment with tirzepatide (Table 1). These findings suggest that tirzepatide may provide the additional benefit of mitigating key CMD risk factors such as hyperlipidemia.

Although anti-obesity drugs have been proven to be efficacious, adverse effects have been reported when using these drugs. A previous study found that utilizing GLP-1 agonist medications for weight loss was associated with an increased risk of pancreatitis, gastroparesis, and bowel obstruction [35]. A previously published randomized control trial found that 78.9–81.8% of people receiving tirzepatide reported at least one non-serious adverse effect such as nausea, vomiting, diarrhea or constipation. Overall, tirzepatide has demonstrated a safety profile similar to other pharmaceutical agents used to treat obesity [36, 37]. Given that those with SCI often experience changes in bowel and bladder functioning following injury, it is paramount to understand the impact that dual GLP and GIP-1 agonists may have on the gastrointestinal (GI) and genitourinary (GU) systems in those with SCI. While no adverse GU effects were noted by participants who received tirzepatide for 72 total weeks, there were several GI adverse effects reported in the non-SCI clinical trial [34]. After 6 months of treatment with tirzepatide our patient notably did not report any adverse GI effects such as diarrhea or constipation. This is particularly significant for individuals with SCI as they often have established bowel programs tailored to achieve optimal stool consistency.

In addition to minimal adverse GI effects, our patient reports a significant improvement in his dietary cravings, noting that he no longer craves calorie dense foods that he previously craved, such as chocolate cake and alcoholic beverages. Craving less calorie dense foods will help foster sustained weight loss and reduce one’s risk of regaining body weight. Additionally, tirzepatide’s once weekly subcutaneous injections are convenient for patients and likely to foster long-term compliance.

This case report shows promising results with respect to utilizing tirzepatide to treat neurogenic obesity in SCI, however larger-scale studies are needed. A potential limitation of our study is the lack of appropriate pre and post treatment laboratory studies. Although our patient did demonstrate improvements in his lipid profile, obtaining baseline lipid and inflammatory marker levels pre- and post- treatment would provide greater insights into the role of tirzepatide in limiting other CMD risk factors. Additionally, our patient did not receive treatment for one month while hospitalized for a thermal injury; however, it is unclear if that impacted weight loss results. Our patient’s dietary and exercise program adherence is based upon self-report and may be subject to self-report bias. Incorporating the use of food diaries and exercise logs prospectively in future studies will provide objective measures that will limit the likelihood of self-report bias.

## Conclusion

The metabolic dysfunction associated with SCI and barriers to adequate exercise for weight loss place individuals with SCI at increased risk for obesity and CMD. Tirzepatide may be a practical, safe, and effective adjunct therapy to exercise and dietary intervention to help facilitate weight loss and prevent CMD in those with SCI. Further research is indicated to examine the long-term efficacy, benefits, and adverse effects that may be associated with tirzepatide. To the author’s knowledge, this is the first case report of tirzepatide being used to treat obesity associated with SCI.

## Data Availability

Data collected can be found within the published article, and they are also available from the corresponding author on reasonable request.
